# Impact of long-term antiretroviral therapy on gut and oral microbiotas in HIV-1-infected patients

**DOI:** 10.1038/s41598-020-80247-8

**Published:** 2021-01-13

**Authors:** Mayumi Imahashi, Hirotaka Ode, Ayumi Kobayashi, Michiko Nemoto, Masakazu Matsuda, Chieko Hashiba, Akiko Hamano, Yoshihiro Nakata, Mikiko Mori, Kento Seko, Masashi Nakahata, Ayumi Kogure, Yasuhito Tanaka, Wataru Sugiura, Yoshiyuki Yokomaku, Yasumasa Iwatani

**Affiliations:** 1 Clinical Research Center, National Hospital Organization Nagoya Medical Center, Nagoya, Aichi Japan; 2 Department of HIV Clinic, National Hospital Organization Nagoya Medical Center, Nagoya, Aichi Japan; 3 Division of Basic Medicine, Nagoya University Graduate School of Medicine, Nagoya, Aichi Japan; 4 Department of Virology, Nagoya City University, Nagoya, Aichi Japan; 5 Department of Gastroenterology and Hepatology, Kumamoto University, Kumamoto, Kumamoto Japan; 6 Present Address: Graduate School of Environmental and Life Science, Okayama University, Okayama, Japan

**Keywords:** Retrovirus, Clinical microbiology, Microbiome

## Abstract

In HIV-1-infected patients, antiretroviral therapy (ART) is a key factor that may impact commensal microbiota and cause the emergence of side effects. However, it is not fully understood how long-term ART regimens have diverse impacts on the microbial compositions over time. Here, we performed 16S ribosomal RNA gene sequencing of the fecal and salivary microbiomes in patients under different long-term ART. We found that ART, especially conventional nucleotide/nucleoside reverse transcriptase inhibitor (NRTI)-based ART, has remarkable impacts on fecal microbial diversity: decreased α-diversity and increased ß-diversity over time. In contrast, dynamic diversity changes in the salivary microbiome were not observed. Comparative analysis of bacterial genus compositions showed a propensity for *Prevotella*-enriched and *Bacteroides*-poor gut microbiotas in patients with ART over time. In addition, we observed a gradual reduction in *Bacteroides* but drastic increases in *Succinivibrio* and/or *Megasphaera* under conventional ART. These results suggest that ART, especially NRTI-based ART, has more suppressive impacts on microbiota composition and diversity in the gut than in the mouth, which potentially causes intestinal dysbiosis in patients. Therefore, NRTI-sparing ART, especially integrase strand transfer inhibitor (INSTI)- and/or non-nucleotide reverse transcriptase inhibitor (NNRTI)-containing regimens, might alleviate the burden of intestinal dysbiosis in HIV-1-infected patients under long-term ART.

## Introduction

Improvement in anti-HIV-1 drugs and treatment strategies has reduced mortality and increased the life expectancy of HIV-1-infected patients^1,2^. The conventional regimen of antiretroviral therapy (ART) contains two nucleotide/nucleoside reverse transcriptase inhibitors (NRTIs) and a key drug of either an integrase strand transfer inhibitor (INSTI), a non-nucleotide reverse transcriptase inhibitor (NNRTI), or a protease inhibitor (PI), although INSTIs are currently the most recommended key drugs. However, because ART does not cure HIV-1 infection, life-long ART is essential for patients. Therefore, increased caution is fundamental against the emergence of side effects and/or long-term toxicities of anti-HIV-1 drugs to maintain patients’ healthy statuses. One of the major side effects is gastrointestinal symptoms, such as diarrhea, nausea, and abdominal bloating^3–5^. The side effects are often associated with regimens including an NRTI or a PI^6–8^. Gastrointestinal symptoms such as nausea, diarrhea, vomiting, and abdominal pain are also observed, though infrequently, after preexposure prophylaxis (PrEP) with NRTI(s)^3–5,9^. Previous studies have suggested that in addition to sexual behavior and/or HIV-1 infection, ART influences the composition of the gut microbiota in HIV-1-infected patients^10–16^. Comparative analyses between the gut microbiomes have shown that the compositions are changed before and after starting ART^15,17–19^. In addition, it has been reported that the microbiotas in body sites other than the gut, such as the oral microbiota, are also changed under ART^19,20^. Furthermore, in vitro studies have indicated that an NRTI, zidovudine (AZT), exhibits antibacterial effects^21^ likely due to inhibition of bacterial polymerases and/or induction of the SOS DNA damage response^22–25^. This evidence indicates that the gut microbiota of HIV-1-infected patients may be linked to gastrointestinal defects even under short-term ART. However, it is not fully understood how long-term ART changes the microbiota of individual patients over time and whether different ART regimens have diverse impacts on the gut and oral microbiotas in HIV-1-infected patients. Here, to probe the long-term effects of ART on the commensal microbiota in patients, we performed microbial ribosomal RNA (rRNA) gene sequencing of the fecal and salivary microbiomes of HIV-1-infected patients and compared time-course changes in microbial diversity. Furthermore, we analyzed the potential effects of different regimens on the gut and oral microbial populations over time.

## Results

### Demographics of this study cohort

A total of 20 HIV-1-infected Japanese patients were enrolled in this study: 6 NRTI(+), 9 NRTI(–)PI(–), and 5 NRTI(–)PI(+). Of the 20 patients, 18 were men who have sex with men (MSM), and two were males [NRTI(–)PI(–)] with heterosexuality or unknown sexuality based on their medical interviews (Supplementary Tables S1 and S2). Viral loads in the NRTI(+) group declined to an undetectable level (20 copies/mL) by 24 weeks after ART initiation, whereas those in the NRTI(–) groups were undetectable or close to the undetectable level for 24 weeks. In the NRTI(+) group, the ART regimen was an INSTI (dolutegravir, DTG or raltegravir, RAL) with two NRTIs (Supplementary Table S1), whereas that in the NRTI(–)group was an INSTI with an NNRTI [NRTI(–)PI(–)] or a PI with either maraviroc (MVC) or an INSTI [NRTI(–)PI(+)] (Supplementary Table S2). In this study, thirteen sets of healthy donor (HD) samples were also collected as controls. The CD4^+^ T cell counts in the NRTI(+) group were lower than those in the NRTI(–) group, although there were no statistically significant differences [P = 0.051 and P = 0.057 for NRTI(–)PI(–) and NRTI(–)PI(+), respectively]. In contrast, the ages in the NRTI(+) group were younger than those in the NRTI(–) group [P = 0.028 and P = 0.026 for NRTI(–)PI(–) and NRTI(–)PI(+), respectively] (Table 1).

**Table 1:** Demographics of the patients at their recruitments.

	HD	NRTI(+)	NRTI(–)PI(–)	NRTI(–)PI(+)
Gender	Male (n = 8) Female (n = 5)	Male (n = 6)	Male (n = 9)	Male (n = 5)
Age (year)	33.1 ± 7.4	37.5 ± 7.0	53.3 ± 13.6	49.2 ± 6.1
CD4^+^ T-cell counts (cells/μL)^a^	ND	288 ± 115	526 ± 235	520 ± 198
ART regimen	NA	INSTI + NRTI (n = 6)	INSTI + NNRTI (n = 9)	INSTI + PI/r (n = 2) PI/r + MVC (n = 3)
Treated periods^b^	NA	0 week (n = 6)	> 2 year (n = 5) 1–2 year (n = 4)	> 2 year (n = 3) 1–2 year (n = 2)
Sample collection^c^	NA	0 week 1 week 12 weeks 24 weeks	x weeks x + 12 weeks	x weeks x + 12 weeks

The numbers in parentheses indicate the numbers of healthy donors (HD) or patients.

ND, not determined: NA, not applicable: INSTI, integrase strand transfer inhibitor: NNRTI, non-nucleoside reverse transcriptase inhibitor: PI/r, Ritonavir-Boosted Protease Inhibitor: MVC, Maraviroc.

^a^Avg ± SD.

^b^The treated periods before the recruitment are shown. For the NRTI(–)PI(–) and NRTI(–)PI(+) groups, the periods under NRTI-sparing regime are indicated.

^c^The time points of sampling after ART initiation are shown.

### Impacts of ART on microbiome diversity in HIV-1-infected patients

**Figure 1:**
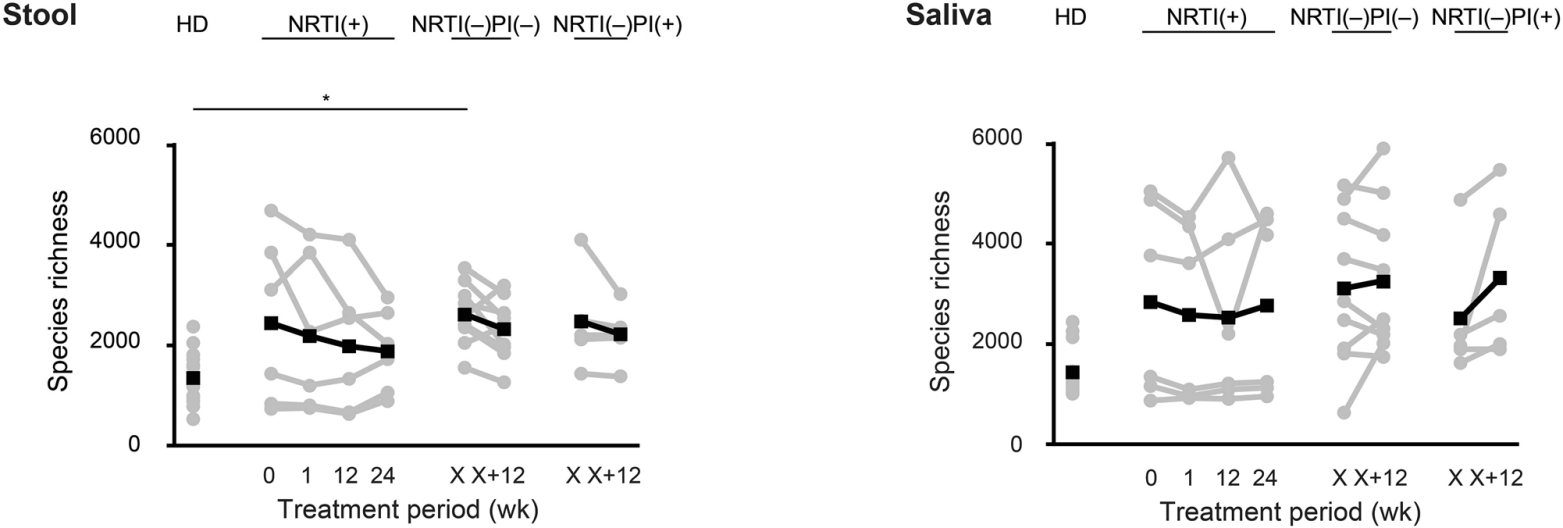
The α-diversities of the fecal and salivary microbiomes. Per sample, > 5000 filtered sequences were analyzed in the healthy donor (HD) (n = 13), NRTI(+) (n = 6), NRTI(–)PI(+) (n = 9), and NRTI(–)PI(–) (n = 5) groups. The patients’ samples were collected at 0, 1, 12, and 24 weeks after the treatment for NRTI(+) and at 12 weeks after switching to NRTI(–)PI(–) or NRTI(–)PI(–) at a certain time point, x weeks. The α-diversity estimates based on the Chao1 index were plotted with gray circles and lines. The averages are represented in black circles and lines. *P < 0.05.

A total of > 5000 filtered sequences per sample were obtained from fecal and salivary samples using deep sequencing. To understand whether ART changes the overall diversity of the gut or oral microbiota in patients, we initially analyzed the species-richness estimates (Chao1 indices) of the microbiome, which display microbial α-diversity within individuals. As shown in Fig. 1, the average species richness of the fecal microbiome was higher in the three groups than in the HD group, especially in the NRTI(–)PI(–) group (P = 0.04, x weeks), although there was individual variability (Fig. 1). In contrast, such a significant difference was not observed in the salivary microbiomes. The results suggest that the gut and salivary microbial α diversities are higher in the HIV-1-infected patients with NRTI(–)PI(–) than in uninfected donors in our cohorts. Comparative analyses of the α-diversities with each parameter indicate that the fecal microbial diversities are correlated with the salivary diversity (r = 0.63, P < 0.0001) (Supplementary Fig. S1A). However, the diversities were not correlated with CD4+ T cell counts or age, among patients’ samples (Supplementary Fig. S1B). Interestingly, the average diversity over time appeared to decrease in the fecal microbiota but not in the salivary microbiota of the patients during conventional NRTI-based ART (Fig. 1). Moreover, we noticed that the fecal α-diversity in the NRTI(–) groups was inversely correlated with the duration of NRTI treatment (before switching to NRTI-sparing ART, P = 0.052) but not with the duration of NRTI-sparing treatment (P = 0.82) (Supplementary Fig. S1). These results suggest that conventional NRTI-based ART exerts a suppressive impact on microbial α-diversity in the gut but not in the saliva over time.

**Figure 2:**
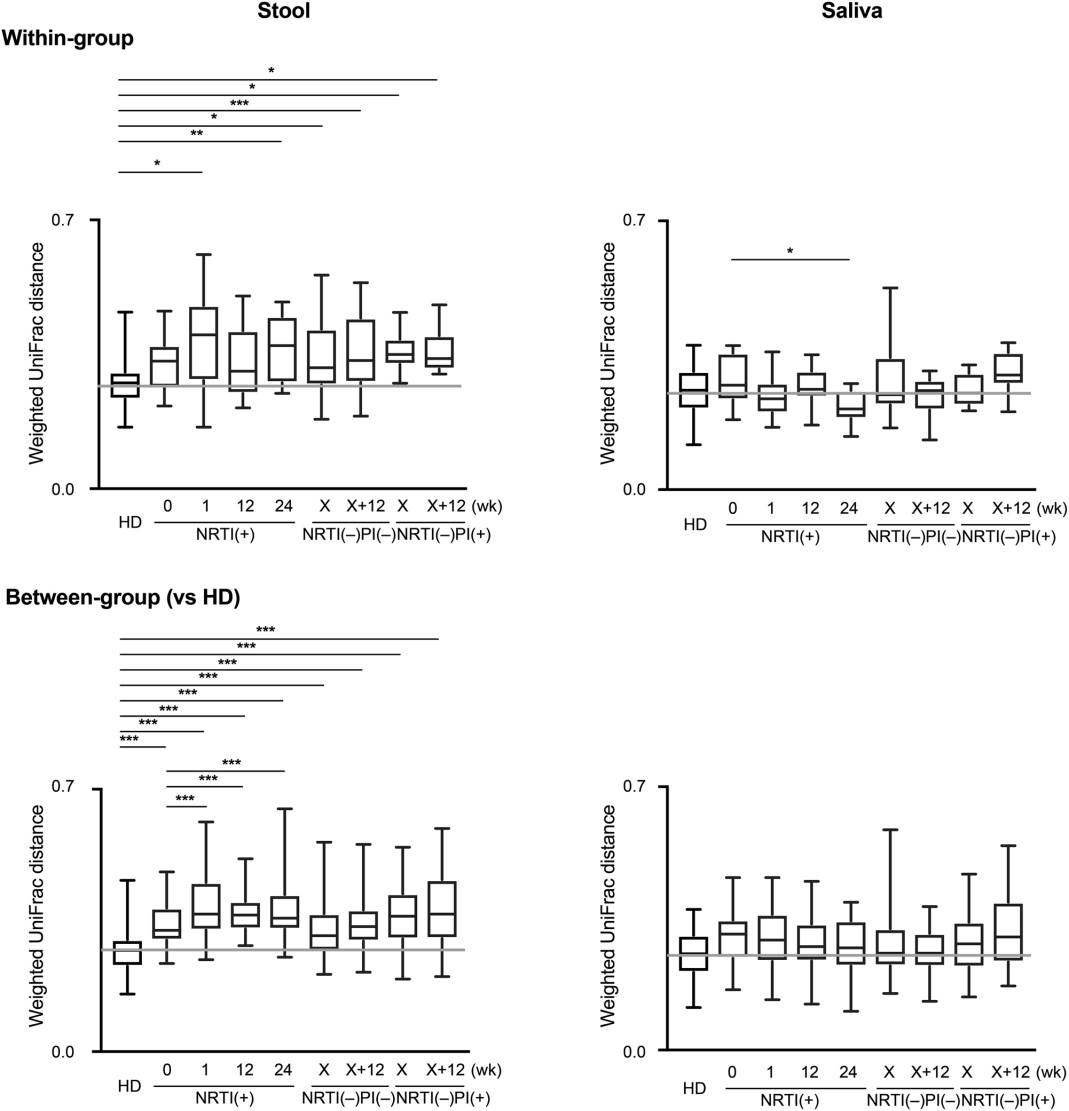
The ß-diversities of the fecal and salivary microbiomes. The quantitative ß-diversities, evaluated using weighted UniFrac distances, were compared among the healthy donor (HD) (n = 13), NRTI(+) (n = 6), NRTI(–)PI(+) (n = 9), and NRTI(–)PI(–) (n = 5) groups. The ß-diversities of two samples within a group (within-group) or between groups (between-group) are represented with box and whisker plots. The median values of ß-diversities in HDs are highlighted with gray horizontal lines. *P < 0.05, **P < 0.01, ***P < 0.001.

Next, to compare microbial diversities between individuals, we analyzed the quantitative ß-diversities based on the weighted UniFrac distances (WUDs)^26^. In fecal microbiomes, measurements of the UniFrac distance levels within each group (within-group) demonstrated that the ß-diversities were significantly increased in the patients compared with those in the HDs (0.2707), especially in the NRTI(+) samples (0.3691 at 24 weeks) and in the NRTI(–)PI(–) and NRTI(–)PI(+) samples, 0.3295 and 0.3453 at x + 12 weeks, respectively (Fig. 2). The diversity analyses between the HD group and each patient group (between-group) also showed that fecal microbial ß-diversities were significantly higher in the patient groups than in the HD group (Fig. 2). In sharp contrast, the salivary microbial ß-diversities appear to be similar between the HD and the patient groups. These findings suggest that regardless of the treatment regimen, ART increases the gut microbial ß-diversity more significantly than the oral microbial diversity in HIV-1-infected patients, who have intrinsically high diversity.

### Influence of ART on genus proportions of microbiomes

**Figure 3:**
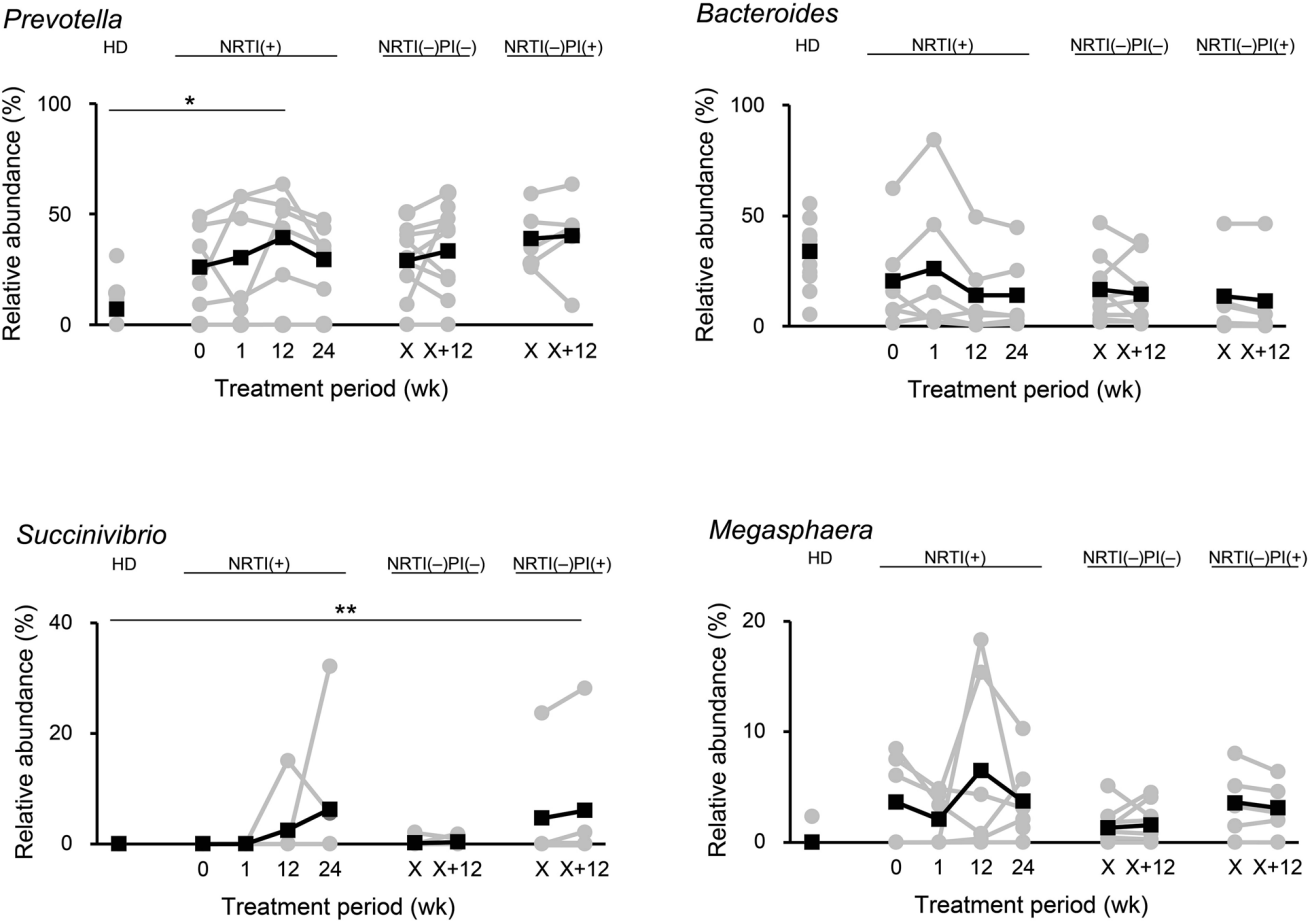
Temporal changes in the relative abundances of fecal genera in HIV-1-infected patients. The percentage (%) of each genus relative to the total relative abundance (%) was calculated in the samples of the healthy donor (HD) (n = 13), NRTI(+) (n = 6), NRTI(–)PI(+) (n = 9), and NRTI(–)PI(–) (n = 5) groups. Gray and black plots represent each sample and the averages, respectively. *P < 0.05, **P < 0.01.

We next analyzed the effects of ART on the compositions of the fecal and salivary microbiomes in the patients. A total of 135 family and 288 genus 16S rRNA gene sequences present at more than 0.5% abundance in any given samples were obtained from our microbiome samples through Illumina deep sequencing. These > 0.5% genus populations are equivalent to more than 25 reads per sample in our obtained Illumina sequence data because > 5000 filtered sequences for each sample were analyzed. Of the microbial families and genera, we investigated microbial populations whose proportions were significantly different in relative abundance between the HD group and any one of the three patient groups at a given sampling time point and then compared the microbial composition patterns among the donor groups (Table 2 and Supplementary Table S3). As shown in Table 2, the results demonstrated that the overall composition patterns of the fecal microbiomes were largely distinct between HIV-1-infected patients and the HDs, as previously reported^12–15,27,28^. In stool, the microbiomes displayed *Prevotella* enrichment and *Bacteroides* depletion in all of the HIV-1-infected patient groups compared with those in the HD group (Fig. 3). Importantly, we found that the proportions of *Prevotella* were significantly increased by 12 weeks in the NRTI(+) group (P = 0.03 compared to HD). In contrast, *Bacteroides* abundance tends to be reduced by 12 weeks in the NRTI(+) group (P = 0.08 compared to 0 week in the NRTI(+)). These propensities were relatively weak in the two NRTI(–) groups. These results suggest that ART, especially NRTI-based ART, is likely to exaggerate the propensity for a *Prevotella*-enriched and *Bacteroides-*poor gut microbiota over time in the patients. These differences in the fecal microbiomes of the patients were in contrast to those in the salivary microbiomes. Three major genera in the salivary microbiomes, *Prevotella*, *Streptococcus*, and *Veillonella*^18,19,29^, were not largely differentially abundant between the patients and HDs (Fig. S2). Of note, fecal *Faecalibacterium*, and *Lachnospiraceae* were also observed as minor genera that differed between the HIV-1-infected and HD groups (Table 2).

**Table 2:** Microbial profiles at the genus level in healthy donors and HIV-1-infected patients.

	Family	Genus	Relative abundance (%)								
			HD	NRTI(+)				NRTI(–)PI(–)		NRTI(–)PI(+)	
				0 week	1 week	12 week	24 week	X week	X + 12 week	X week	X + 12 week
Stool	*Bacteroidaceae*	*Bacteroides*	33.3	20.3	26.0	13.9	13.9	16.5	14.5	13.7	11.6
	*Prevotellaceae*	*Prevotella*	7.4	26.2	30.5	39.3*	29.4	29.1	33.2	38.8	40.3
	*Rikenellaceae*	Unclassified	1.0	0.3	0.3	0.3	0.6	0.5	0.5	0.1	0.1
	*S24-7*	Unclassified	0.1	0.7	0.6	0.6	0.8	0.3	0.1	0.0	0.0
	*[Barnesiellaceae]*	Unclassified	1.2	0.3	0.3	0.2	0.3	0.3	0.4	0.0*	0.3
	*[Paraprevotellaceae]*	*[Prevotella]*	0.3	5.6	3.5	3.6	2.8	4.3	4.6	4.7	4.4
	*Lachnospiraceae*	Other	2.0	1.3	1.0	1.2	1.0	0.6	0.6	1.1	0.7
		Unclassified	6.6	3.3	2.6	2.8	3.0	6.5	6.0	5.6	4.3
		*Blautia*	3.1	2.0	1.1	1.7	1.4	1.5	1.1*	0.8*	1.3
		*Lachnospira*	4.1	0.7	0.4	0.4	0.5	2.4	1.5	0.7	0.2
		*[Ruminococcus]*	0.5	0.6	0.3	0.7	0.3	0.3	0.2	0.2	0.3
	*Ruminococcaceae*	Unclassified	13.7	4.7	2.6*	2.7*	5.0	6.3	5.6	2.6*	2.5*
		*Faecalibacterium*	2.0	0.4	0.2*	0.3	0.3	1.3	0.6	0.5*	0.3*
		*Oscillospira*	0.6	0.9	0.6	0.4	0.6	0.4	0.4	0.1	0.4
		*Ruminococcus*	0.2	0.8	0.8	0.9	0.8	0.2	1.3	0.0	0.2
	*Veillonellaceae*	*Acidaminococcus*	0.3	1.3	0.9	1.9	1.4	1.4	1.2	1.9	1.5
		*Dialister*	0.7	1.6	2.0	1.3	2.2	1.7	1.7	2.7	3.3
		*Megasphaera*	0.2	3.7	2.1	6.5	3.8	1.3	1.6	3.6	3.1
		*Mitsuokella*	0.0	0.9	0.9	0.5	0.6	2.5	3.3*	0.9	1.4
		*Phascolarctobacterium*	2.1	1.2	0.4	1.6	1.0	1.9	1.8	0.8	1.2
	*Erysipelotrichaceae*	*Catenibacterium*	0.0	0.9	0.4	2.0	0.8	0.4	0.6	0.6	0.8
		*[Eubacterium]*	0.1	0.5*	0.5	0.4	0.6	0.6	0.2	0.2	0.2
	*Fusobacteriaceae*	*Fusobacterium*	0.6	1.9	8.5	0.5	2.7*	5.4	4.5	1.2	0.1
	*Alcaligenaceae*	*Sutterella*	1.7	3.0	1.1	2.2	2.6	0.9	0.7	1.1	1.2
	*Succinivibrionaceae*	*Succinivibrio*	0.0	0.0	0.0	2.5	6.3	0.2	0.3	4.7	6.1**
	*Enterobacteriaceae*	Unclassified	0.7	0.1	0.1	0.0	0.1	0.4	1.2	1.0	1.0
Saliva	*Micrococcaceae*	*Rothia*	2.2	4.8	3.8	4.2	2.1	3.5	2.9	3.8	4.7
	*Coriobacteriaceae*	*Atopobium*	0.9	1.3	2.4	1.1	2.3	1.0	1.1	1.2	1.3
	*Prevotellaceae*	*Prevotella*	20.1	26.1	26.2	24.6	31.6	14.9	16.2	22.4	23.5
	*[Paraprevotellaceae]*	*[Prevotella]*	8.2	2.2*	3.3	3.9	3.8	4.7	5.9	4.2	3.3
	*Flavobacteriaceae*	*Capnocytophaga*	1.3	0.7	0.6	1.1	0.7	1.5	1.7	1.7	1.5
	*Streptococcaceae*	*Streptococcus*	9.7	16.8	20.0	16.3	15.8	9.3	9.7	17.7	16.6
	*Lachnospiraceae*	Unclassified	0.3	0.4	0.3	0.7	0.5	0.4	0.6	0.2	0.2
	*Fusobacteriaceae*	*Fusobacterium*	5.4	3.1	3.2	5.1	3.8	6.2	8.6	8.6	10.5
	*Burkholderiaceae*	*Lautropia*	0.6	0.2	0.1	0.3	0.2	0.1	0.3	0.3	0.2
	*Neisseriaceae*	*Neisseria*	10.6	3.6	3.1	4.6	2.1	8.1	8.2	4.3	3.2
	*Campylobacteraceae*	*Campylobacter*	1.7	3.6	2.5	2.1	2.6	2.3	1.9	1.2	1.0
	*Pasteurellaceae*	*Haemophilus*	9.2	5.4	4.7	5.0	4.6	6.0	6.5	4.2	2.9*

The asterisks represent statistical significances, compared with the values of healthy donor (HD) group. * P < 0.05, **P < 0.01.

As the second most prominent feature of the fecal microbiota in the infected patients, the proportions of rare genera, *Succinivibrio* and *Megasphaera,* were high, with considerable variation between individuals, whereas they were under the detection level in the HD group, with one exception for *Megasphaera* (Fig. 3). Two patients (MB102 and MB105) in the NRTI(+) group had undetectable levels of *Succinivibrio* before ART, whereas the levels were drastically increased up to > 10% at 12 and 24 weeks after ART initiation. In addition, the proportions of *Succinivibrio* for one (MB218) NRTI(–)PI(+) patient were significantly increased during ART, while those for 14 other patients in the NRTI(–) groups were only marginally increased. There were no significant clinical signs that were reported by the patients with a high abundance of *Succinivibrio*. Furthermore, *Megasphaera* abundance was not significantly elevated during ART in patients, although it was increased from 0% (1 week) to > 15% (12 weeks) in two NRTI(+) patients. These results suggest that ART may affect two genera, *Succinivibrio* and *Megasphaera*, rarely found in the gut microbiota, although this effect appears considerably variable among patients. There were no significant differences in the other fecal microbial proportions between the patient groups and HD group, such as *Clostridium*, a well-known genus associated with antibiotic-associated diarrhea^30^, and periodontal-disease-related genera, *Porphyromonas*, *Actinomyces*, *Treponema*, *Aggregatibacter*, *Shuttleworthia*, *Gemella*, *Dialister*, and *Granulicatella*.

In salivary microbiomes, a notable feature was that the proportions of salivary *[Prevotella]* and *Haemophilus* were relatively lower in patients, regardless of the ART regimen, than in the HDs (Supplementary Fig. S2). Nevertheless, the overall composition patterns of salivary microbiomes were similar between the patients and HDs, which appears to be in contrast to those of the gut microbiomes (Supplementary Fig. S3). In addition, we observed no drastic changes in salivary microbiomes of the patients among the three groups, suggesting that ART exerts less impact against the microbiota in saliva than in the gut.

## Discussion

Several reports have documented the effects of ART on the fecal and salivary microbiomes in HIV-infected patients. However, it has not been fully understood whether different ART regimens have any distinct impacts on the intestinal and oral microbiotas and, if any, how the regimen changes the microbial composition over time. Here, to probe these questions, we investigated fecal and salivary microbiomes in patients with NRTI-based ART or NRTI-sparing ART through analysis of bacterial 16S rRNA gene sequences using deep sequencing. First, our analysis of microbial α-diversity suggested that ART, especially conventional NRTI-based ART, may have a suppressive impact on fecal microbial diversity within individuals over time. In addition, ART, regardless of regimen, tends to increase the fecal microbial ß-diversity in HIV-1-infected patients, indicating that the gut microbiota varies between patients. In contrast, such drastic changes in α-diversity and ß-diversity were not observed in the salivary microbiomes of the patients. These results suggest that NRTI-based ART has a higher impact on the gut microbiota than NRTI-sparing ART and contributes to microbial imbalance, dysbiosis, in the gut but not in the mouth. Comparative analysis of bacterial genus compositions showed a propensity for a *Prevotella*-enriched and *Bacteroides*-poor microbiota in the gut in patients with ART over time. In addition, the proportions of rare genera, *Succinivibrio* and *Megasphaera*, were considerably high only in the stool of patients treated with NRTIs or PIs, although their magnitudes were different between individuals. These results suggest that long-term ART, especially NRTI-including therapy, has more suppressive impacts on microbiota composition and variability in the gut than in the mouth, which potentially causes intestinal dysbiosis in HIV-1-infected patients. Furthermore, NRTI-sparing ART, especially with INSTI and NNRTI regimens, might alleviate a burden on intestinal dysbiosis in infected patients under long-term ART.

Previous studies have demonstrated that the intestinal microbial alterations previously ascribed to HIV-1 infection may also be associated with sexual preference^13^. HIV-seronegative MSM often have a distinct fecal microbiota composition, with elevated microbial diversity and enrichment in *Prevotella*, which is independent of HIV-1 status^13,31^. Similarly, our patient cohort, mainly MSM, also had *Prevotella*-rich and *Bacteroides*-depleted fecal microbiotas, with elevated microbial diversity between individuals, which was comparable with that of the uninfected HD group. Importantly, our time-course analysis of the cohort’s microbiotas indicated that ART, especially NRTI-based ART, exacerbates imbalanced conditions of the intestinal microbiota. In this prospective observational study, all the recruited patients with NRTI-sparing regimens had relatively long histories of NRTI-based ART (2–17 years) before switching to their NRTI-sparing ART (> 1 year) because it is difficult to recruit treatment-naïve patients who plan to take ART without NRTIs at their initial diagnosis point. Nevertheless, we observed fecal α-diversity reduction in the NRTI(–) groups, which was correlated with only the duration of NRTI-including ART before switching to NRTI-sparing ART (Supplementary Fig. S1). These new data also suggest that long-term NRTI-based ART impacts intestinal microbial composition in patients. Several previous studies suggest that microbial dysbiosis in infected patients may cause intestinal mucosal dysfunction and/or barrier vulnerability and increase translocation of bacteria into the systemic periphery^32^, which may lead to HIV pathogenesis as well as clinical disorders, including inflammatory bowel diseases^33^. In this study, however, we found no obvious clinical symptoms that were correlated with such intestinal dysbiosis in the patients for half of a year. Further investigations are necessary to understand the pathogenesis driven by intestinal dysbiosis in HIV-1-infected patients under NRTI-based ART.

In the context of specific microbial genera, the proportions of *Succinivibrio* or *Megasphaera* were significantly enriched in NRTI(+) groups compared with those in the NRTI(–) groups: high *Succinivibrio* abundance was found in *Prevotella*-rich microbiomes, whereas increased *Megasphaera* abundance was found in *Bacteroides*-rich microbial populations (Supplementary Fig. S4). This result suggests that there may be a certain combinatorial correlation between genera during dysbiosis that may be driven by NRTI-based ART. Previous studies suggest that *Succinivibrio* and *Megasphaera* may be associated with defects in gastrointestinal functions, such as diarrhea and abdominal pain^6–8,34^. Antibiotic-induced diarrhea is known to be related to an increase in intestinal succinate^35–38^, which is mainly produced by the *Succinivibrio* genus^39,40^. As NRTIs potentially have antibacterial effects^21–25^, the NRTI-based regimen may increase succinate production in the gut. Similarly, *Megasphaera* may cause excessive gas (CO_2_ and H_2_) production that results in abdominal pain and/or other gastrointestinal symptoms (e.g., nausea)^41,42^. In this study, however, it is difficult to assess whether such NRTI-driven increases in *Succinivibrio* or *Megasphaera* are solely responsible for gastrointestinal side effects because most of the patients in the NRTI(+) group (MB102, MB105, MB107, and MB108) suffered diarrhea before ART (Supplementary Table S1). Therefore, further analyses are required to clarify the association between these genera and gastrointestinal defects driven by NRTIs. Of note, we characterized bacteria in the *Succinivibrio* and *Megasphaera* genera at species-level resolution through a BLAST search of Oxford Nanopore long-read sequences (Supplementary Fig. S5), although taxonomic assignment at the species level was technically difficult due to ambiguity among the sequences of these strains. The results indicated that sequences classified as the *Succinivibrio* genus were similar to those of *Succinivibrio dextrinosolvens* (identity ~ 96%), while those within the *Megasphaera* genus were similar to those of either *Megasphaera elsdenii* or *Megasphaera massiliensis* (identities ~ 98%) (Supplementary Fig. S5). These results suggest that these particular species might be involved in gastrointestinal side effects in the NRTI(+) group over a long period of ART.

In summary, we investigated the time-course dynamics of the fecal and salivary microbiomes in HIV-1-infected patients receiving ART using deep sequencing. The results indicated significant microbial dysbiosis in the gut but not in saliva in the patients over a long period of ART. This propensity was observed in the NRTI(+) group more significantly than in the NRTI-sparing group. In particular, the prominent features were reduced α-diversity, elevated ß-diversity and drastic changes in the proportions of *Bacteroides*, *Succinivibrio*, and *Megasphaera* in the gut. There were no significant gastrointestinal side effects in our study period, although further investigation is required for the assessment of HIV-1-infected people’s quality of life (QOL) in the current long-term ART era. These studies help us to understand how to succeed in the life-long health maintenance of these patients.

## Methods

### Approval of clinical research

The study was approved by the ethics committee at the Nagoya Medical Center (registration #2014-776) and conducted according to the principles expressed in the Declaration of Helsinki. Written informed consent for the collection of samples and subsequent analyses was obtained from all the participants.

### Sample collection

Patients who visited the National Hospital Organization Nagoya Medical Center in Japan since September 2014 were recruited. The study was approved by the ethics committee at the Nagoya Medical Center (registration #2014-776) and conducted according to the principles expressed in the Declaration of Helsinki. Written informed consent for the collection of samples and subsequent analyses was obtained from all the participants. The exclusion criteria for participation were patients who had suffered or were suffering from inflammatory bowel disease, who received transrectal drug administration within 48 h, or who had taken antibiotics within 30 days. We limited recruitment to only Japanese HIV-1-infected males because of low incidences of HIV-1 infection among females in Japan^43,44^ and of different pharmacology of NRTIs between men and women^45,46^. In addition, the nationality was limited to Japanese because it is known that there are some biased effects of ethnicity and geography on the gut microbiota^16,47–51^. Stool and saliva samples were collected by using a stool collection kit (TechnoSuruga Laboratory Co., Ltd.) and Oragene DISCOVER kit (DNA Genotek), respectively. Samplings were requested from two groups of patients: (1) those receiving conventional ARTs containing an NRTI [NRTI(+) group] at 0, 1, 12, and 24 weeks after ART initiation (Supplementary Table S1) and (2) those receiving an NRTI-sparing regime at a certain time point (x weeks) and 12 weeks after x weeks (x + 12 weeks) [NRTI(–) group]. The latter group was further divided into two groups, NRTI(–)PI(+) and NRTI(–)PI(–), based on the use of PIs because PIs are strongly associated with gastrointestinal adverse effects^6–8^ (Supplementary Table S2). In this observational study, as the NRTI(–) group, we recruited patients who had been undergoing ART with an NRTI-sparing regimen for > 1 year. These patients had changed the ART from conventional NRTI-based to an NRTI-sparing regimen due to emergence of side effects or entry in a clinical trial (detailed in supplementary Table S2). We also collected samples from HD volunteers.

### Deep sequencing with Illumina MiSeq

Microbial DNAs were extracted from the fecal and salivary samples using a DNeasy PowerSoil Kit (Qiagen) and an Oragene purifier (DNA Genotek), according to their manufactures’ instructions. We subsequently prepared 16S rRNA gene amplicons for Illumina MiSeq deep sequencing according to the 16S metagenomic sequencing library preparation guide (Illumina). Briefly, the V3–V4 region of 16S rRNA genes was amplified by PCR, followed by appending the indices and Illumina sequencing adaptors at the ends using a Nextera XT Index Kit (Illumina). The resultant gene amplicons were subjected to deep sequencing on an Illumina MiSeq system using MiSeq Reagent Kits v3 (2 × 300 bp paired-end) (Illumina).

Microbiome data analyses were performed based on the obtained paired-end read sequences, basically with the MACQIIME v1.9.1 package^52,53^. First, each set of paired-end reads was merged to generate long error-corrected sequences using the PEAR v0.9.6 program^54^, which shows superior performance as a merging program^55^. Next, quality filtering at Phred score > 25 was performed with MACQIIME, and then, chimeric sequences were removed with usearch8.1.1861^56^. Taxonomic assignments of bacterial family and genus and estimations of α-/ß-diversities were achieved using MACQIIME from > 5000 of the filtered non-chimeric sequences per sample. The α-diversity was calculated based on multiple metrics of Chao1 indices that represent species richness, whereas quantitative ß-diversities were evaluated according to the WUDs^26^. Phylogenetic trees were also constructed by the unweighted pair group method with arithmetic mean (UPGMA) method.

### Analyses of sequencing data

To assign taxonomic classification of each sequence at the species level, we performed a BLAST search with NCBI BLAST 2.9.0 + (ftp://ftp.ncbi.nlm.nih.gov/blast/executables/blast+/LATEST/) against the 16S rRNA sequence data set in the DNA Data Bank of Japan (DDBJ) (ftp://ftp.ddbj.nig.ac.jp/ddbj_database/16S/). Anomalistically, the relative prevalence of the 16S rRNA sequences only in the *Megasphaera* genus was estimated based on the read numbers in each monophyletic clade because the sequences of some species in the genus are similar to each other. The clades of *Megasphaera* are defined by our phylogenetic tree analyses via maximum likelihood estimation methods with 500 bootstrap replicates in MEGA X (https://www.megasoftware.net/).

### Statistical tests

Clinical data were statically examined with Student’s *t*-test. For unpaired or paired non-parametric tests on paired or unpaired data of microbial sequences, multiple comparisons and post-hoc tests were performed. We performed the non-parametric Spearman’s rank-order test to evaluate the significance of the correlation coefficient. These tests were performed using PRISM7 (GraphPad, San Diego, USA).

## Supplementary Information

Supplementary Information.

## Data Availability

The sequence data reported are available in the DNA Data Bank of Japan (DDBJ) Sequenced Read Archive under BioProject accession ID PRJDB9093.
