# Supplementary material for: Impact of long-term antiretroviral therapy on gut and oral microbiotas in HIV-1-infected patients
Source: Sci Rep. 2021 Jan 13;11:960. doi: 10.1038/s41598-020-80247-8 (PMC7806981; doi:10.1038/s41598-020-80247-8)
Supplement: Supplementary file 1 — Supplementary Information. [file 41598_2020_80247_MOESM1_ESM.pdf]

**Impact of long-term antiretroviral therapy on gut and oral microbiotas in HIV-1-infected patients**

Mayumi Imahashi[1,2], Hirotaka Ode[1], Ayumi Kobayashi[1], Michiko Nemoto[1,†], Masakazu Matsuda[1], Chieko Hashiba[2], Akiko Hamano[1], Yoshihiro Nakata[1,3], Mikiko Mori[1,2,3], Kento Seko[4], Masashi Nakahata[2], Ayumi Kogure[2], Yasuhito Tanaka[4,5], Wataru Sugiura[1], Yoshiyuki Yokomaku[1,2], & Yasumasa Iwatani[1,3]*

[1]Clinical Research Center, National Hospital Organization Nagoya Medical Center, Nagoya, Aichi, Japan, [2]Department of HIV clinic, National Hospital Organization Nagoya Medical Center, Nagoya, Aichi, Japan, [3]Division of Basic Medicine, Nagoya University Graduate School of Medicine, Nagoya, Aichi, Japan, [4]Department of Virology, Nagoya City University, Nagoya, Aichi, Japan, [5]Department of Gastroenterology and Hepatology, Kumamoto University, Kumamoto, Kumamoto, Japan

**Supplementary Information**

**Supplementary Figure S1**: Correlation of bacterial α-diversities in the fecal (Stool) or salivary (Saliva) microbiomes.

**Supplementary Figure S2**: Temporal changes in the relative abundances of salivary genera in HIV-1-infected patients.

**Supplementary Figure S3**: UPGMA phylogenetic tree representing the relationship among samples.

**Supplementary Figure S4**: Phylogenetic tree of species in the Megasphaera genus.

**Supplementary Figure S5**: Time-course changes in the relative abundance of fecal species in HIV-1-infected patients.

**Supplementary Table S1**: Demographics of the patients in the NRTI(+) group

**Supplementary Table S2**: Demographics of patients in the NRTI(–) groups

**Supplementary Table S3**: Microbial family-level profiles of health donors and HIV-1-infected patients

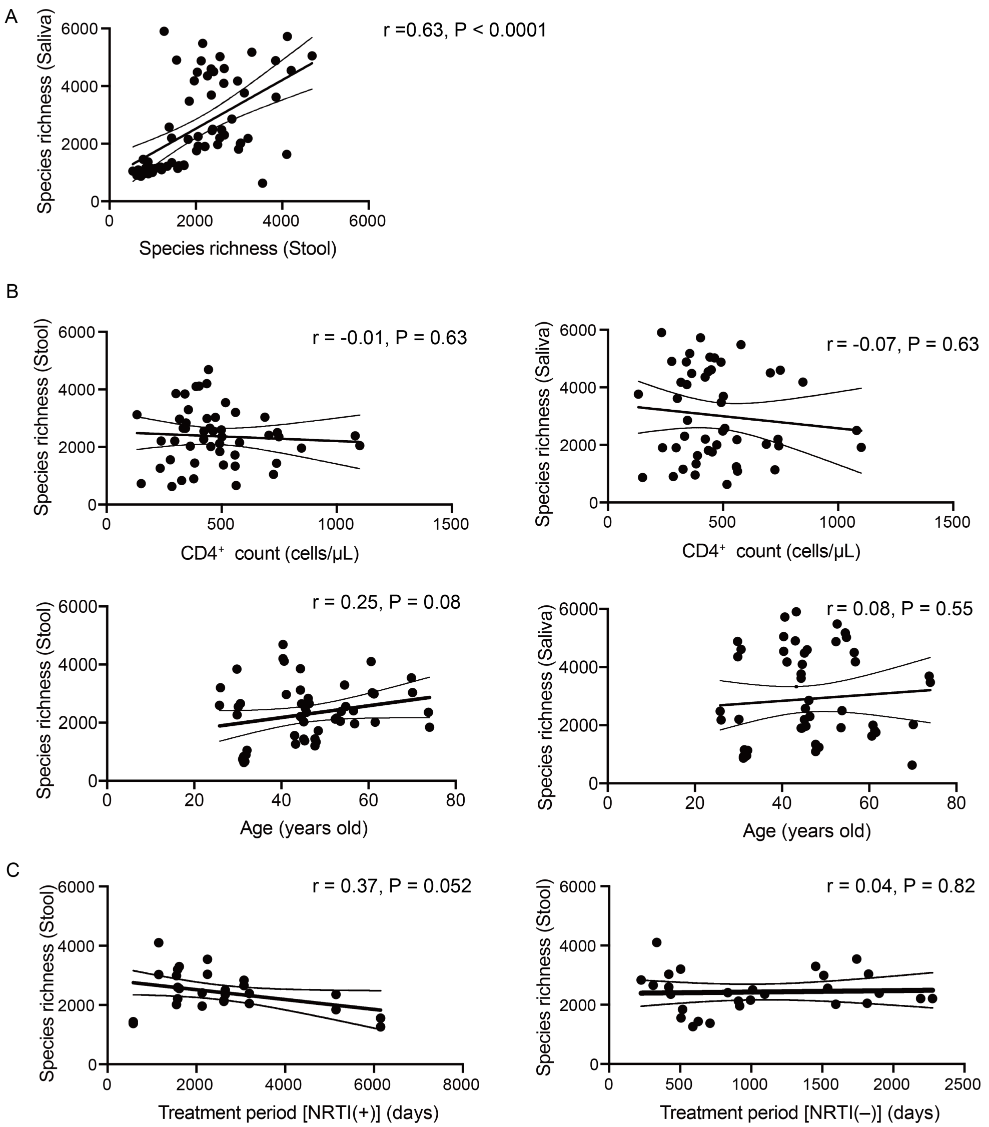


**Figure S1.** Correlation of bacterial α-diversities in the fecal (Stool) or salivary (Saliva) microbiomes. (A) The α-diversities were compared among the fecal and salivary microbiomes in all the samples analyzed in this study. The diversities are represented as "species richness". (B) The α-diversities of the fecal and salivary microbiomes are plotted against $CD4^+$ T cell counts at the corresponding time points. (C) The fecal α-diversities are plotted against the duration (days) of NRTI-based (left) and NRTI-sparing (right) treatments in the NRTI(–) groups. In each graph, a linear regression line and its 95% confidence interval are also shown.

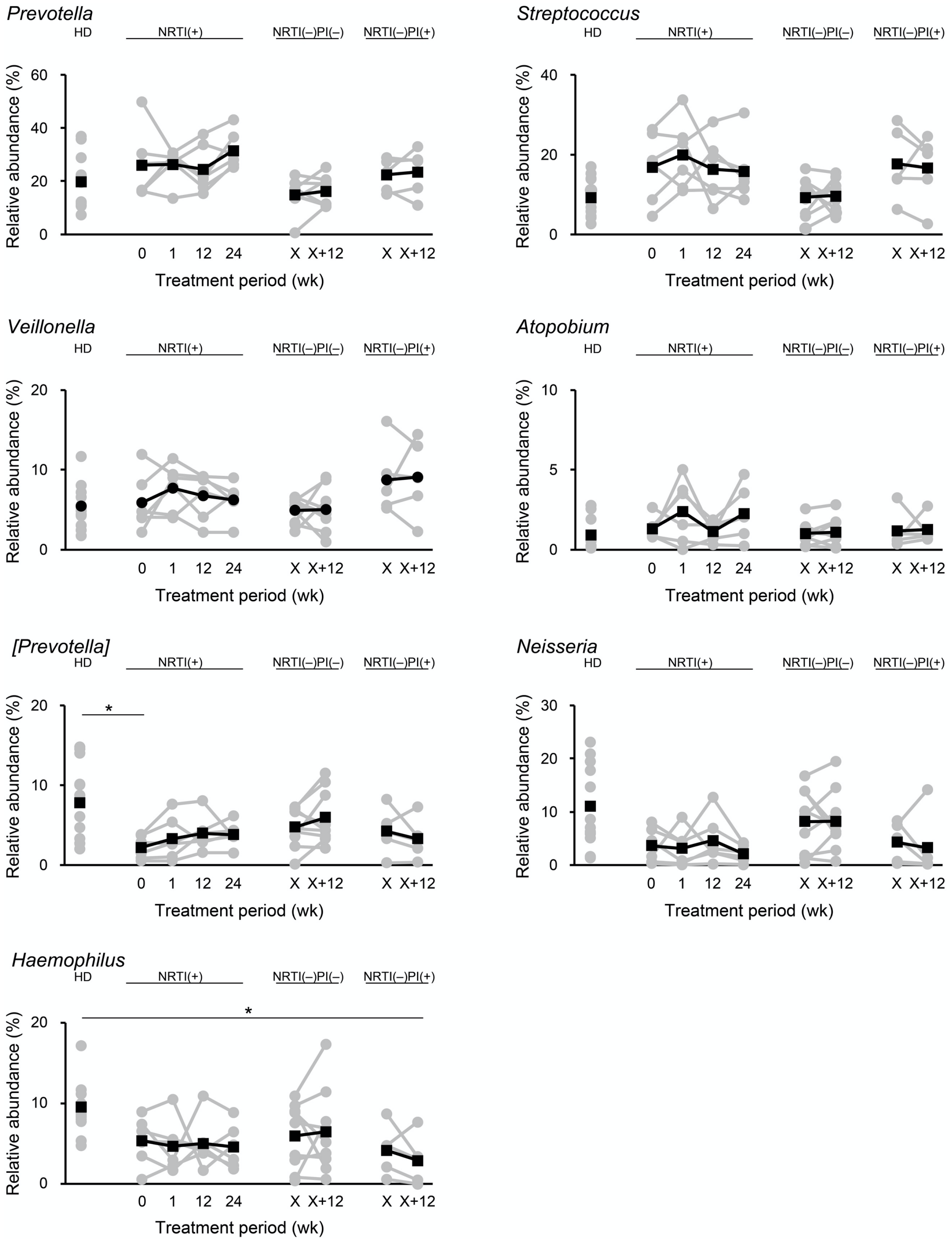


**Figure S2.** Temporal changes in the relative abundances of salivary genera in HIV-1-infected patients. The percentage (%) of each genus relative to the total, shown as “relative abundance (%)”, was calculated. Graphs show the relative abundances of *Prevotella*, *Streptococcus*, *Veillonella*, *Atopobium*, *[Prevotella]*, *Neisseria*, and *Haemophilus* in the healthy donor (HD), NRTI(+), NRTI(–)PI(–),NRTI(–)PI(+) groups. Gray and black plots represent each sample and the averages, respectively. * $P < 0.05$.

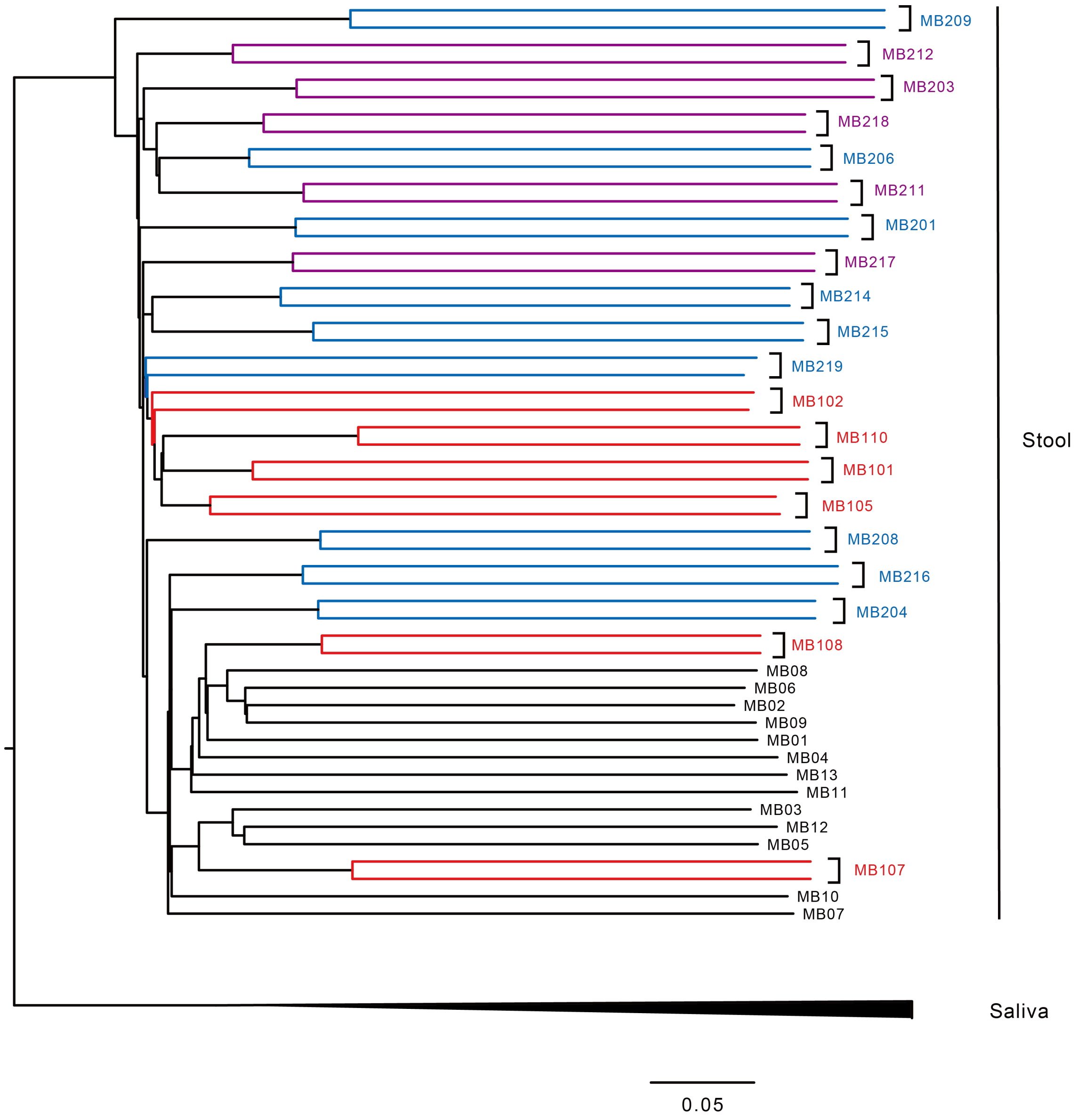


**Figure S3.** UPGMA phylogenetic tree representing the relationship among samples. Sequence data at 0 and 24 wks in NRTI(+) samples were used to construct the UPGMA tree. The fecal microbiomes of the healthy donor (HD), NRTI(+), NRTI(–)PI(–) and NRTI(–)PI(+) groups are shown in green, red, blue, and purple, respectively. Notably, fecal microbiomes of the HDs are *Bacteroides* rich, whereas those of the MSMs are *Prevotella* rich.

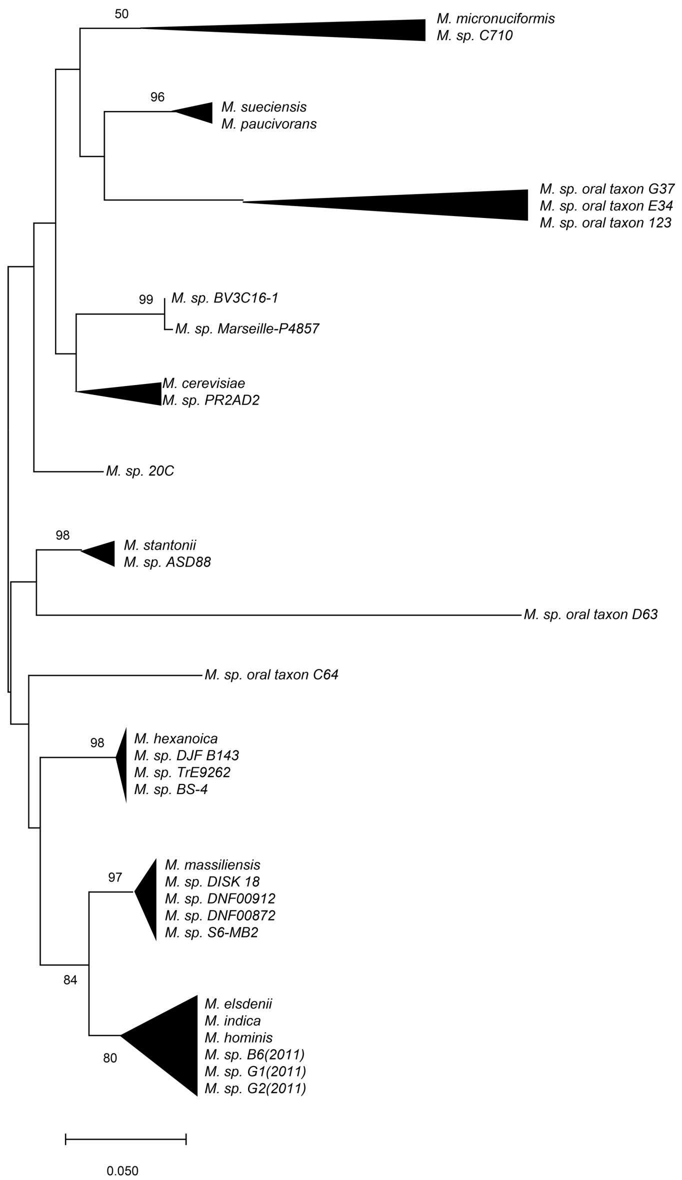


**Figure S4.** Phylogenetic tree of species in the *Megasphaera* genus. The tree was constructed using the 16S rRNA gene data of the *Megasphaera* registered in the DDBJ (ftp://ftp.ddbj.nig.ac.jp/ddbj_database/16S/).

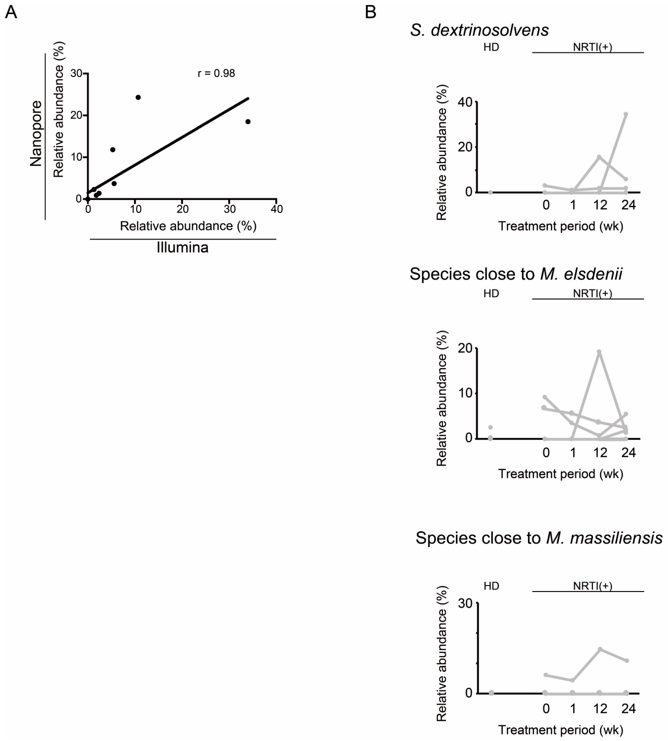


**Figure S5.** Time-course changes in the relative abundance of fecal species in HIV-1-infected patients. (A) The relative abundance data of *S. dextrinosolvens*, *M. elsdenii*, and *M. massiliensis* were compared between the Illumina MiSeq and Oxford Nanopore results. Nanopore sequencing was performed on a MinION using R9.4.1 flow cells (Oxford Nanopore Technologies). Base calling of the raw Nanopore sequencing data was subsequently achieved with GUPPY v3.1.5 (Oxford Nanopore Technologies). The graph shows the plots of their relative abundances obtained from the two platforms. The representative data in the graph are shown for fecal samples of the NRTI(+) group at 24 wks (Spearman's rank-order correlation coefficient, r = 0.98, P < 0.0001). (B) The relative abundance (%) in each sample based on the Illumina MiSeq sequencing was plotted. The graphs show the relative abundances (%) of *S. dextrinosolvens*, *M. elsdenii*, and *M. massiliensis* in the healthy donor (HD) and NRTI(+) groups. The numbers of *M. elsdenii* and *M. massiliensis* include their related species. Gray and black plots represent each sample and the averages, respectively. Because some species in the *Megasphaera* genus are phylogenetically close to each other (Fig. S4), the relative abundances of the *Megasphaera* genus were scored as the same species that fall into a monophyletic clade.

**Table S1.** Demographics of the patients in the NRTI(+) group

| Patient ID | Gender | Transmission route | Information at entry (treatment naïve) | | | ART regimen | Diarrhea before ART |
|---|---|---|---|---|---|---|---|
| | | | Age (yr) | $CD4^+$ cell (cells/uL) | Viral load (copies/mL) | | |
| MB101 | M | Homo | 30 | 340 | 4630 | TDF/FTC+DTG | No |
| MB102 | M | Bi | 40 | 443 | 527 | TDF/FTC+DTG | Yes |
| MB105 | M | Homo | 44 | 132 | 165000 | TDF/FTC+DTG | Yes |
| MB107 | M | Homo | 31 | 282 | 63100 | DVY+DTG | Yes |
| MB108 | M | Homo | 31 | 150 | 196000 | TRI | Yes |
| MB110 | M | Homo | 48 | 383 | 36700 | DVY+DTG | No |

DVY, Descovy (Emtricitabine/Tenofovir alafenamide)

TRI, Triumeq (DTG/Abacavir/Lamivudine)

**Table S2.** Demographics of patients in the NRTI(–) groups

NRTI(–)PI(–) group

| Patient ID | Gender | Transmission route | Information at entry | | | | ART | | | History of administered NRTI |
|---|---|---|---|---|---|---|---|---|---|---|
| | | | Years after diagnosis | Age (yr) | CD4 count (cells/uL) | VL (copies/ mL) | NRTI-Sparing Regimen | Reasons | Years after diagnosis | |
| MB201 | M | Hetero | 8 | 56 | 705 | < 20 | DTG+RPV | Osteoporosis | 6 | TDF/FTC, ABC/3TC |
| MB204 | M | Unknown | 15 | 73 | 501 | < 20 | RAL+ETR | Lipoatrophy | 14 | TDF+AZT+ 3TC, ABC/3TC+ TDF |
| MB206 | M | Homo | 8 | 54 | 355 | < 20 | RAL+ETR | Flatulent colic | 4 | ABC/3TC, AZT/3TC |
| MB208 | M | Homo | 9 | 46 | 345 | < 20 | DTG+RPV | Lipoatrophy | 8 | AZT/3TC, TDF/FTC |
| MB209 | M | Homo | 18 | 43 | 277 | < 20 | DTG+RPV | Hepatic toxicity | 17 | ddI+ABC, ABC |
| MB214 | M | Homo | 11 | 69 | 517 | < 20 | DTG+RPV | Anemia | 6 | ABC/3TC |
| MB215 | M | Bi | 14 | 53 | 1100 | < 20 | RAL+ETR | Drug eruption | 9 | TDF+3TC |
| MB216 | M | Bi | 8 | 61 | 436 | < 20 | DTG+RPV | Cough | 4 | ABC/3TC, d4T+3TC |
| MB219 | M | Homo | 5 | 25 | 499 | < 20 | DTG+RPV | Lipoatrophy | 4 | d4T+3TC, TDF/FTC |

| Patient ID | Gender | Transmission route | Information at entry | | | | ART | | | History of administered NRTI |
|---|---|---|---|---|---|---|---|---|---|---|
| | | | Years after diagnosis | Age (yr) | CD4 count (cells/uL) | VL (copies/ mL) | NRTI-Sparing Regimen | Reasons | Years after diagnosis | |
| MB203 | M | Homo | 10 | 45 | 742 | < 20 | LPV/r+MVC | Clinical trail | 7 | TDF/FTC |
| MB211 | M | Homo | 10 | 52 | 491 | 21.3 | DRV/r+MVC | Clinical trail | 7 | AZT/3TC |
| MB212 | M | Homo | 3 | 45 | 739 | < 20 | DRV/r+MVC | Lipoatrophy | 2 | TDF/FTC |
| MB217 | M | Bi | 4 | 60 | 389 | 25 | DRV/r+DTG | Cardiomyopathy | 3 | ABC/3TC, 3TC |
| MB218 | M | Bi | 10 | 44 | 237 | < 20 | DRV/r+DTG | Bone marrow suppression | 4 | TDF/FTC, TDF+ABC |

< 20, undetectableVL, viral load

RPV, Rilpivirine

ETR, Etravirine

LPV, Lopinavir

DRV/r, Darunavir/Ritonavir

**Table S3.** Microbial family-level profiles of health donors and HIV-1-infected patients

Stool

| Family | HD | NRTI(+) | | | | NRTI(–)PI(–) | | NRTI(–)PI(+) | |
|---|---|---|---|---|---|---|---|---|---|
| | | 0 wk | 1 wk | 12 wk | 24 wk | X wk | X+12 wk | X wk | X+12 wk |
| *Prevotellaceae* | 7.4 | 26.2 | 30.5 | 39.3* | 29.4 | 29.1 | 33.2 | 38.8 | 40.3 |
| *[Barnesiellaceae]* | 1.2 | 0.3 | 0.3 | 0.2 | 0. 3 | 0.3 | 0.4 | 0.0* | 0.3 |
| *Ruminococcaceae* | 16.6 | 6.8 | 4.2* | 4.3* | 6.7 | 8.2 | 7.8 | 3.2* | 3.4* |

Relative abundance (%) applies to all value columns.

Saliva

| Family | HD | NRTI(+) | | | | NRTI(–)PI(–) | | NRTI(–)PI(+) | |
|---|---|---|---|---|---|---|---|---|---|
| | | 0 wk | 1 wk | 12 wk | 24 wk | X wk | X+12 wk | X wk | X+12 wk |
| *[Paraprevotellaceae]* | 8.2 | 2.2* | 3.3 | 3.9 | 3.8 | 4.7 | 5.9 | 4.2 | 3.3 |

The asterisks represent the statistical significances, compared with the values of healthy donor (HD) group. * $P < 0.05$
